# A New Pencil‐Tailed Tree Mouse *Chiropodomys wandingensis* sp. nov. (Rodentia: Muridae) From Yunnan, China

**DOI:** 10.1002/ece3.73991

**Published:** 2026-07-20

**Authors:** Yi‐shun Qian, Xin Mou, Shuo Liu, Song Li

**Affiliations:** ^1^ Kunming Natural History Museum of Zoology, Kunming Institute of Zoology Chinese Academy of Sciences Kunming Yunnan China; ^2^ Key Laboratory of National Forestry and Grassland Administration on Biodiversity Conservation on the Qinghai‐Xizang Plateau, Kunming Institute of Zoology Chinese Academy of Sciences Kunming Yunnan China; ^3^ Yunnan Key Laboratory of Biodiversity Information, Kunming Institute of Zoology Chinese Academy of Sciences Kunming Yunnan China

**Keywords:** *Chiropodomys*, molecular biology, morphology, new species, Yunnan

## Abstract

The genus *Chiropodomys* currently has six valid species recorded globally, widely distributed in Southeast Asia. No new *Chiropodomys* species has been described since the 1980s. In this study, a single specimen collected from Wanding, Yunnan Province, China, was analyzed using a combined methodology of morphological comparative analysis and molecular biology. The results show that the specimen is not only distinct from all known species of *Chiropodomys* in external morphology, but also the genetic distance between it and its close relatives is at least 13.06% (Cyt *b*) and 10.40% (COI). Based on these results, it is proved that the specimen represents a new *Chiropodomys* species: *C*. *wandingensis* sp. nov. Furthermore, based on this finding and in conjunction with literature data, a key to the species within the genus was compiled.

## Graphical abstract

The genus *Chiropodomys* has seen no new species described since the 1980s. Through integrated morphological and molecular analysis, a specimen from Yunnan, China, is identified as a distinct new species, *C. wandingensis* sp. nov., supported by significant genetic divergence from its closest relatives.

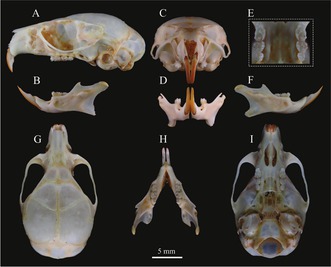

## 1 Introduction

Peters (1869) established the genus *Chiropodomys* Peters, 1869 with *Chiropodomys penicillatus* Peters, 1869 as the type species. Musser (1979), based on his examination of the holotype of *C. penicillatus* (BZM 3476), established that it is conspecific with *Chiropodomys gliroides* (Blyth, 1856) from the Malay Peninsula, and therefore treated the former as a subspecies of the latter. Additionally, he emphasized that despite *C. penicillatus* being a junior synonym at the species level, its nomenclatural status as the type species (by original designation) of the genus *Chiropodomys* remains unchanged. Consequently, the type species of *Chiropodomys* was subsequently designated as *C. gliroides* (Blyth 1856). The genus *Chiropodomys* currently contains six valid species: *C. gliroides* , *C. major* , *C. pusillus* , *C. karlkoopmani* , *C. muroides* , and *C. calamianensis* (Musser and Carleton 2005; Wilson et al. 2017). *C. gliroides* exhibits the widest geographic range within the genus, extending from the Indochinese Peninsula to the Indo‐Malayan Archipelago (Musser and Carleton 2005).

Thomas (1893) described *Chiropodomys pusillus* Thomas, 1893 from Mount Kinabalu, Malaysia; however, Musser (1979) later reclassified it as a subspecies of *C. gliroides* , and Musser and Carleton (2005) observed that *C. pusillus* is smaller than *C. gliroides* and exhibits greater size variation, noting its distribution across multiple regions of Borneo. Gerrie and Kennerley (2016) reported its occurrence in Sabah, Sarawak, and southern Kalimantan. *Chiropodomys major* Thomas, 1893 was initially described from Sarawak (Thomas 1893). Musser (1979) subsequently expanded its distribution to Sarawak and Sabah in Malaysian Borneo, while Gerrie and Kennerley (2017) suggested it may extend further into Kalimantan and other parts of Borneo. Allen (1927) described *Chiropodomys fulvus* Allen, 1927 based on specimens from Yunnan, China, but Tate (1936) questioned its placement within *Chiropodomys*, while Ellerman (1941) accepted Allen's classification; the taxonomic debate was ultimately resolved by Anthony (1941), who erected the new genus *Vernaya* Anthony, 1941 and designated *C. fulvus* as its type species. Taylor (1934) originally described *Insulaemus calamianensis* (Taylor 1934) from Busuanga Island, Calamian Islands, Philippines; Sanborn (1952) later transferred this species to *Chiropodomys*, and Musser (1979) recorded its distribution within the Palawan Faunal Region for *Chiropodomys calamianensis* (Taylor 1934), and Kennerley (2016) subsequently reported additional records from Dumaran Island and Calauit in the Philippines. Medway (1965) described the new species *Chiropodomys muroides* Medway, 1965 based on a specimen from Bundu Tuhan, Mount Kinabalu, Sabah, Malaysia. Musser and Carleton (2005) noted that although *C. muroides* has been documented only from Gunung Kinabalu (Malaysia) and North Kalimantan (Indonesia), it may have a broader distribution across Borneo. Musser (1979) first documented *Chiropodomys karlkoopmani* Musser, 1979 from North Pagai Island, but Jenkins and Hill (1982) later restricted its known distribution to Siberut Island, Indonesia; however, subsequent studies have confirmed its occurrence on both the Pagai and Siberut islands (Musser and Carleton 2005; Clayton and Kennerley 2016).

Wu and Deng (1984) described *Chiropodomys jingdongensis* Wu and Deng, 1984 as a new species from Jingdong, Yunnan. However, Musser and Carleton (2005) later treated *C. jingdongensis* as a synonym of *C. gliroides* , arguing that it differs only in slightly larger average values for a few cranial measurements, with no other notable distinctions. However, this conclusion was based solely on limited morphological comparisons and lacked support from molecular data.

Since 1984, no additional new species of this genus have been reported. However, this may not indicate that the species diversity of the genus has been completely exhausted, but rather that traditional taxonomy based on morphology has difficulty in recognizing cryptic diversity.

Wilson et al. (2017) summarized the characteristics of *Chiropodomys* as follows: the body is slender, covered with short, soft and dense fur mixed with scattered, barely visible guard hairs, a short head, large eyes, medium‐sized ears and long, narrow whiskers, the hind feet are short and broad, with a nailed hallux on the first toe and short, curved claws on the second to fourth toes, the tail is long and well‐furred with a distinct terminal tuft. *Chiropodomys* species are arboreal rodents with specialized grasping hands and feet (Weiss et al. 2020).

While sorting specimen collections at the Kunming Natural History Museum of Zoology, Kunming Institute of Zoology, Chinese Academy of Sciences, we found one specimen that closely matches the morphological characteristics of *Chiropodomys* but shows clear differences from the six currently recognized valid species of this genus. So, the study uses morphological and molecular methods to perform taxonomic analysis of this specimen.

## 2 Materials and Methods

### 2.1 Material Collection

*Chiropodomys* sp.1 (field number KIZ20220150) was collected on July 10, 2019, from Wanding Town, Ruili City, Dehong Dai and Jingpo Autonomous Prefecture, Yunnan Province (24.11° N, 98.12° E; altitude: 1174 m), in a subtropical mountain forest. The specimen is stored in the Kunming Natural History Museum of Zoology, Kunming Institute of Zoology, Chinese Academy of Sciences.

Specimen with collection number KIZ20250092 was collected on July 20, 2025, from Caijia Village, Wenlong Town, Jingdong County, Pu'er City, Yunnan Province (100.64° E, 24.60° N). Its morphological characteristics are consistent with the original description of *Chiropodomys jingdongensis* provided by Wu and Deng (1984). According to Wu and Deng (1984), all type specimens of *C. jingdongensis* were initially deposited at the Ecological Department of Kunming Branch, Chinese Academy of Sciences, and were later transferred to the specimen collection of the Kunming Natural History Museum of Zoology, Kunming Institute of Zoology, Chinese Academy of Sciences. However, after careful verification, we were unable to locate the aforementioned type specimens in this collection.

### 2.2 Morphological Analyses

According to the morphological taxonomic methods, the detailed description and comparison of pelage characteristics are performed.

**FIGURE 1:**
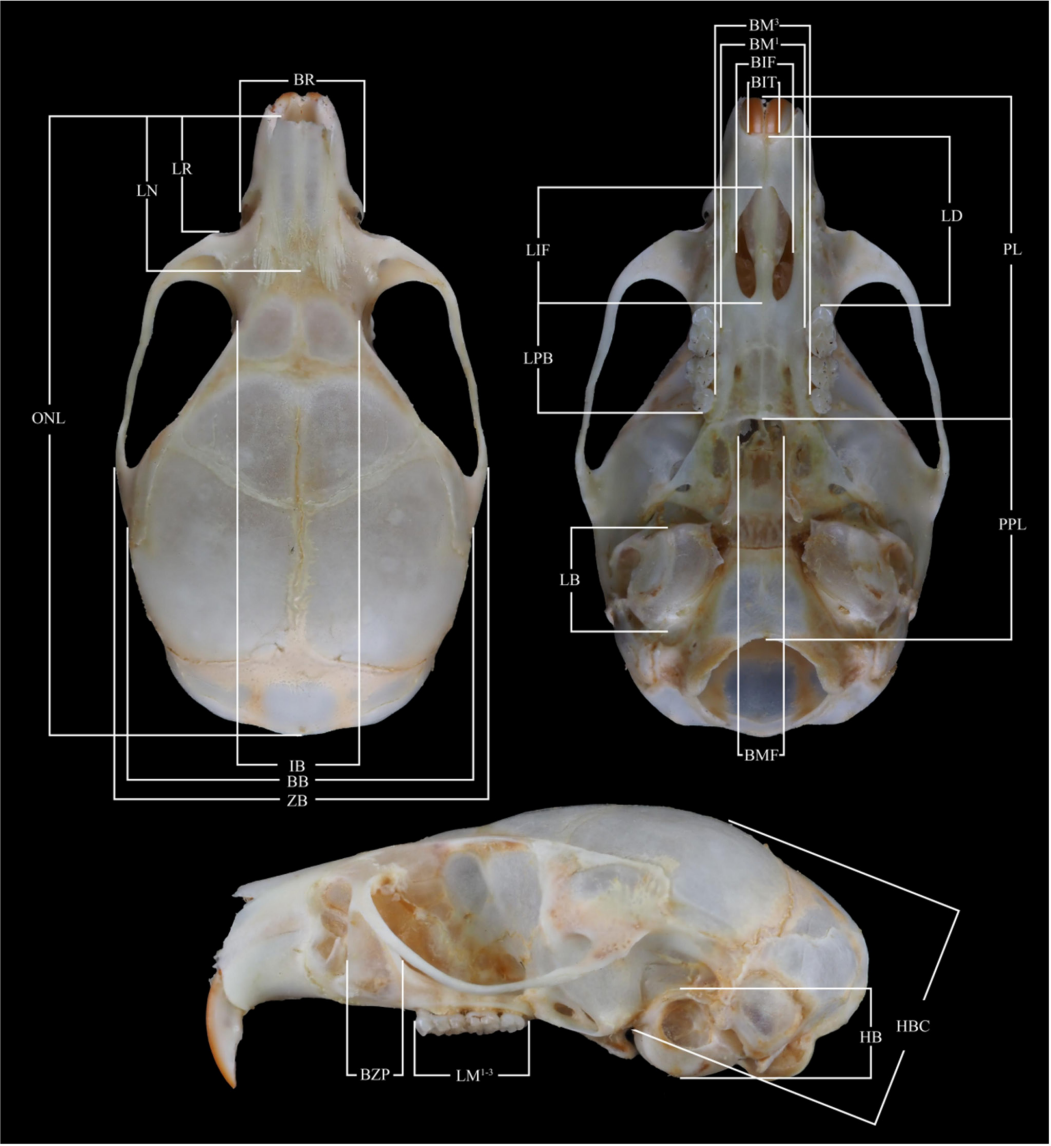
Schematic diagram of craniometric measurements of the skull.

All cranial measurements followed the standards established by Musser (1979), with specific measurement definitions as follows: **HBL**: head‐body length, from front of mouth to anus; **TL**: tail length, from anus to tip of tail; **HF**: hindfoot length, from extremity of heel behind os calcis to extremity of longest digit, including claws; **EL**: ear length, from lower border of external auditory meatus to tip of pinna; **ONL**: occipitonasal length, the distance from the tip of the nasals to the posterior margin of the occiput; **ZB**: zygomatic breadth, the greatest breadth across the zygomatic arches; **IB**: interorbital breadth, the least distance, as viewed dorsally, across the frontal bones between the orbital fossae; **LN**: length of nasals, the distance from the anterior tip of the nasal bones to the most posterior suture between the nasal and frontal bones, measured parallel to the surface of the nasals; **LR**: length of rostrum, from the tip of the nasal bones to the posterior margin of the zygomatic notch (the anterior edge of the dorsal maxillary root of the zygomatic plate); **BR**: breadth of rostrum, the greatest breadth across the rostrum, including the bony capsules enclosing the nasolacrimal canals; **BB**: breadth of braincase, measured just above the squamosal roots of each zygomatic arch; **HBC**: height of braincase, from the top of the braincase to the ventral surface of the basisphenoid bone; **BIT**: breadth across incisor tips, the distance across the tips of the incisors; **BZP**: breadth of zygomatic plate, the least distance between the anterior and posterior edges of the zygomatic plate; **LD**: length of diastema, the distance from the posterior alveolar margins of the upper incisors to the anterior alveolar margins of the first upper molars; **PL**: palatal length, the distance from the anterior alveolar margins of the incisors to the posterior edge of the palatal bridge; **PPL**: postpalatal length, the distance from the posterior margin of the palatal bridge to the posterior edge of the basioccipital bone–the ventral lip of the foramen magnum; **LPB**: length of palatal bridge, the distance from the posterior edge of the incisive foramina to the posterior margin of the bony palate; **BM** ^**1**^: breadth of palatal bridge at first molars, the least distance between the lingual edge of the alveolus of the first molar and the lingual edge of the alveolus of the opposite molar; **BM** ^**3**^: breadth of palatal bridge at third molars, the least distance between the lingual edge of the alveolus of the third molar and the lingual edge of the alveolus of the opposite molar; **LIF**: length of incisive foramina, the distance from the anterior edge of one of the foramina to its posterior edge; **BIF**: breadth across incisive foramina, the greatest distance across both foramina; **IF‐M** ^**1**^: incisive foramina to M^1^, the distance from the posterior margins of the incisive foramina to the anterior alveolar margins of the first molars; **BMF**: breadth of mesopterygoid fossa, the distance from one edge of the mesopterygoid fossa to the other; **LB**: length of bulla, the length of the bulla, excluding the bony eustachian tube; **HB**: height of bulla, the distance from the dorsal surface of the bulla to its ventral surface; **LM** ^**1–3**^: alveolar length of maxillary toothrow, the distance from the anterior edge of the alveolus of the first molar to the posterior edge of the alveolus of the third molar (Figure 1). Note: Figure 1 is finished by *Chiropodomys* sp.1, and there is no distance between the incisive foramina and M^1^ in the skull of *Chiropodomys* sp.1, so **IF‐M** ^**1**^ is not labeled.

### 2.3 Phylogenetic Analysis

Phylogenetic analysis was performed based on Cyt *b* and COI genes. Genomic DNA was extracted from muscle tissue samples preserved in absolute ethanol using the Animal Genomic DNA Extraction Kit (200) from Tsingke Biotechnology Co. Ltd. (Beijing, China). The primers used for the amplification of the cytochrome b (Cyt *b*) gene are Molcit‐F (5′‐AATGACATGAAAAATCACCGTTGT‐3′; Ibáñez et al. 2006) and CytB‐H (5′‐CTTTTCTGGTTTACAAGACCAG‐3′; Weyeneth et al. 2008). For the amplification of the cytochrome c oxidase subunit I (COI) gene, the primers employed are BatL5310 (5′‐CCTACTCRGCCATTTTACCTATG‐3′) and R6036R (5′‐ACTTCTGGGTGTCCAAAGAATCA‐3′) (Robins et al. 2007). All sequencing chromatograms were visually inspected and manually corrected to ensure data accuracy prior to phylogenetic analysis.

The phylogenetic trees were constructed using PhyloSuite v1.2.3 (Zhang et al. 2020). Sequence alignment was performed with MAFFT v7.505 (Katoh and Standley 2013). ModelFinder v2.2.0 (Kalyaanamoorthy et al. 2017) was employed to select the optimal substitution model for phylogenetic analysis. Phylogenetic trees constructed based on the Cyt *b* and COI genes both used *Typhlomys chapensis* and *Typhlomys nanus* as outgroups. Maximum likelihood (ML) analysis was conducted using IQ‐TREE v2.2.0 (Nguyen et al. 2015), with the TIM2 + R4 + F model applied to the Cyt *b* dataset and the TIM2 + R2 + F model applied to the COI dataset, both using 1000 standard bootstrap replicates. Bayesian inference (BI) was performed with MrBayes v3.2.7a (Ronquist et al. 2012) under the GTR + I + G + F model for both genes, running two parallel analyses for 5 million generations and discarding the first 25% of samples as burn‐in. The phylogenetic tree was visualized and customized using the Interactive Tree of Life (iTOL) online platform (Letunic and Bork 2024).

Genetic distances for species of *Chiropodomys* were calculated following the grouping of sequences by species. The analysis was conducted using the Kimura 2‐parameter (K2P) model (Kimura 1980) in MEGA v12 (Kumar et al. 2024), with reliability assessed through 1000 bootstrap replicates. Details of the sequences used are provided in Appendix I.

## 3 Results

### 3.1 Morphological Analyses

Based on previous studies (Thomas 1893; Musser 1979), *Chiropodomys* sp.1 is relatively small in body size, smaller than *C. gliroides* , *C. karlkoopmani* , *C. major* , and *C. calamianensis* . Specifically, the HBL of *Chiropodomys* sp.1 is 64.70 mm, while the TL is 91.51 mm. By contrast, the HBL of the latter four species is greater than 80 mm and the TL is greater than 110 mm (Table 1).

**TABLE 1:** Morphological measurements of the genus *Chiropodomys*, all measurements are in millimeters (mm).

	*Chiropodomys* sp.1 KIZ20220150	*C. gliroides* Indochina (Musser 1979)	*C. karlkoopmani* Indomalaya, North Pagai (Musser 1979)	*C. major* Malaysia, Sabah (Musser 1979)	*C. calamianensis* Philippines, Palawan (Musser 1979)	*C. muroides* Malaysia, Sabah (Musser 1979)	*C. pusillus* Malaysia, Sabah holotype (Thomas 1893)
HBL	64.70	90.0 ± 6.3 87.2–92.7 *n* = 21	107	105.3 ± 5.5 102.5–108.1 *n* = 17	117.0 ± 5.9 114.0–120.0 *n* = 4	71.0 ± 7.8 66–80 *n* = 3	76
TL	91.51	115.3 ± 6.8 112.3–118.3 *n* = 21	171	128.4 ± 11.1 123.0–133.8 *n* = 17	153.5 ± 15.5 138.1–168.9 *n* = 4	88.7 ± 3.2 85–91 *n* = 3	81
HF	15.78	19.7 ± 1.1 19.2–20.2 *n* = 21	29	24.1 ± 1.5 23.3–24.9 *n* = 17	25.5 ± 0.6 25.2–25.8 *n* = 4	16.0 ± 1.0 15–17 *n* = 3	15.8
EL	11.36	—	17	16.8 ± 2.9 15.4–18.2 *n* = 17	16.8 ± 1.7 15.0–18.6 *n* = 4	17.0 ± 2.6 14–19 *n* = 3	11.5
ONL	21.25 (damaged)	24.73 ± 0.64 24.34–25.03 *n* = 18	29.3	29.27 ± 0.80 28.89–29.65 *n* = 17	28.73 ± 0.42 28.25–29.21 *n* = 3	20.75 ± 1.20 19.9–21.6 *n* = 2	22.2
ZB	12.56	14.37 ± 0.33 14.21–14.53 *n* = 17	15.7	16.59 ± 0.49 16.35–16.83 *n* = 16	16.4 ± 0.40 15.94–16.86 *n* = 3	11.75 ± 0.64 11.3–12.2 *n* = 2	—
IB	4.34	4.52 ± 0.18 4.44–4.60 *n* = 21	5.7	5.21 ± 0.13 5.15–5.27 *n* = 17	5.33 ± 0.24 5.21–5.45 *n* = 4	4.30 ± 0.20 4.1–4.5 *n* = 3	4.2
LN	5.71 (damaged)	7.47 ± 0.34 7.31–7.63 *n* = 21	9.4	9.75 ± 0.73 9.39–10.11 *n* = 17	9.75 ± 0.19 9.55–9.95 *n* = 4	6.33 ± 0.29 6.0–6.5	7.2
LR	4.15 (damaged)	5.76 ± 0.31 5.62–5.90 *n* = 21	6.7	7.10 ± 0.46 6.88–7.32 *n* = 17	7.05 ± 0.33 6.71–7.39 *n* = 4	4.85 ± 0.35 4.6–5.1 *n* = 2	—
BR	4.07	4.87 ± 0.22 4.77–4.97 *n* = 21	5.9	5.61 ± 0.24 5.49–5.73 *n* = 17	5.78 ± 0.21 5.58–5.98 *n* = 4	4.80 ± 0.00 *n* = 2	—
BB	11.42	12.22 ± 0.23 12.12–12.32 *n* = 18	14.0	13.96 ± 0.26 13.84–14.08 *n* = 17	13.55 ± 0.64 12.65–14.45 *n* = 2	10.65 ± 0.35 10.4–10.9 *n* = 2	11.6
HBC	7.44	7.94 ± 0.29 7.80–8.08 *n* = 17	9.1	9.33 ± 0.33 9.17–9.49 *n* = 17	9.30 ± 0.00 *n* = 2	7.30 ± 0.42 7.0–7.6 *n* = 2	—
BIT	1.23	1.49 ± 0.10 1.45–1.53 *n* = 20	1.7	1.75 ± 0.17 1.67–1.83 *n* = 17	1.78 ± 0.17 1.60–1.96 *n* = 4	1.60 ± 0.00 *n* = 2	—
BZP	2.16	2.51 ± 0.17 2.43–2.59 *n* = 21	3.0	2.75 ± 0.18 2.67–2.83 *n* = 17	3.10 ± 0.16 2.94–3.26 *n* = 4	1.73 ± 0.15 1.6–1.9 *n* = 3	—
LD	5.67	6.46 ± 0.31 6.32–6.60 *n* = 22	8.1	7.84 ± 0.38 7.66–8.02 *n* = 17	7.60 ± 0.14 7.46–7.74 *n* = 4	5.23 ± 0.21 5.0–5.4 *n* = 3	—
PL	10.58	12.47 ± 0.47 12.27–12.67 *n* = 21	14.5	14.92 ± 0.57 14.64–15.20 *n* = 17	14.17 ± 0.32 13.79–14.55 *n* = 3	9.95 ± 0.35 9.7–10.2 *n* = 2	—
PPL	7.54	8.69 ± 0.42 8.49–8.89 *n* = 19	10.1	10.11 ± 0.57 9.83–10.39 *n* = 17	10.15 ± 0.35 9.65–10.65 *n* = 2	7.00 ± 0.42 6.7–7.3	—
LPB	3.68	4.58 ± 0.24 4.48–4.68 *n* = 21	6.7	6.12 ± 0.29 5.98–6.26 *n* = 17	5.78 ± 0.40 5.41–6.33 *n* = 3	4.60 ± 0.28 4.4–4.8 *n* = 2	—
BM^1^	2.82	3.00 ± 0.15 2.92–3.08 *n* = 21	3.3	3.15 ± 0.14 3.09–3.21 *n* = 17	3.20 ± 0.10 3.08–3.32 *n* = 3	2.80 ± 0.00 *n* = 2	—
BM^3^	3.21	3.22 ± 0.20 3.14–3.30 *n* = 21	3.9	3.48 ± 0.14 3.42–3.54 *n* = 17	3.20 ± 0.20 2.96–3.44 *n* = 3	3.10 ± 0.00 *n* = 2	—
LIF	4.02	4.33 ± 0.28 4.21–4.45 *n* = 22	3.3	4.16 ± 0.27 4.02–4.30 *n* = 17	4.33 ± 0.21 4.13–4.35 *n* = 4	2.47 ± 0.25 2.2–2.7 *n* = 2	2.7
BIF	1.90	2.07 ± 0.10 2.03–2.11 *n* = 21	2.3	2.24 ± 0.12 2.18–2.30 *n* = 17	2.28 ± 0.17 2.10–2.46 *n* = 4	1.90 ± 0.00 *n* = 2	—
IF–M^1^	0.00	0.33 ± 0.18 0.25–0.41 *n* = 21	2.0	1.09 ± 0.24 0.97–1.21 *n* = 17	1.08 ± 0.36 0.72–1.44 *n* = 4	1.67 ± 0.15 1.0–1.3	—
BMF	1.49	1.79 ± 0.20 1.71–1.87 *n* = 21	1.9	2.05 ± 0.23 1.93–2.17 *n* = 17	2.07 ± 0.06 2.01–2.13 *n* = 3	1.65 ± 0.21 1.5–1.8 *n* = 2	—
LB	3.56	3.63 ± 0.14 3.57–3.69 *n* = 19	4.1	4.02 ± 0.11 3.96–4.08 *n* = 17	4.15 ± 0.35 3.65–4.65 *n* = 2	3.05 ± 0.07 3.0–3.1 *n* = 2	—
HB	3.33	3.05 ± 0.14 2.99–3.11 *n* = 19	3.8	3.36 ± 0.11 3.30–3.42 *n* = 17	3.45 ± 0.07 3.35–3.55 *n* = 2	2.75 ± 0.07 2.7–2.8 *n* = 2	—
LM^1–3^	3.61	3.92 ± 0.16 3.86–3.98 *n* = 22	4.6	4.72 ± 0.25 4.60–4.84 *n* = 17	4.38 ± 0.17 4.00–4.76 *n* = 4	2.97 ± 0.06 2.9–3.0 *n* = 3	—

*Note:* For species with multiple specimens, the measurements are presented as the mean ± standard deviation, observed range, and sample size.

Although the body size of *Chiropodomys* sp.1 is relatively similar to that of *C. muroides* and *C. pusillus* , there are differences in skull measurements. The BB (11.42 mm), LIF (4.02 mm), LB (3.56 mm), HB (3.33 mm) and LM^1–3^ (3.61 mm) of *Chiropodomys* sp.1 are all larger than those of *C. muroides* (BB: 10.4–10.9 mm, LIF: 2.2–2.7 mm, LB: 3.0–3.1 mm, HB: 2.7–2.8 mm and LM^1–3^: 2.9–3.0 mm). Conversely, the EL (11.36 mm) and LPB (3.68 mm) of *Chiropodomys* sp.1 are smaller than those of *C. muroides* (EL: 14–19 mm; LPB: 4.4–4.8 mm) (Table 1). The dorsal fur of *Chiropodomys* sp.1 is dark brownish‐yellow and the ventral fur is creamy white, the length of the terminal tail tuft is 2–3 mm (Table 2), by contrast, the dorsal fur of *C. muroides* is buffy brown, the ventral fur is buffy dark gray and the tail terminal tuft is 6 mm long (Table 2).

**TABLE 2:** Comparison of external morphological characteristics among *Chiropodomys* sp.1 and other species of the genus *Chiropodomys*.

Species	Body size	Dorsal fur color	Ventral fur color	Tail tuft (mm)
*Chiropodomys* sp.1	Small	Dark brownish‐yellow	Creamy white	2–3
*C. gliroides* ^a^	Large	Pale chestnut	White	4–5
*C. major* ^a^	Relatively large	Grayish brown	White, cream, or buffy white	4–6
*C. pusillus* ^a^	Medium‐sized	Tawny	Pure white	—
*C. karlkoopmani* ^a^	Large	Grayish brown	Pale gray	—
*C. muroides* ^a^	Medium‐sized	Buffy brown	Buffy dark gray	6
*C. calamianensis* ^a^	Very large	Bright buffy brown or chestnut	Orange‐white to orange‐red	6–7

^a^ Data for the comparative species were derived from Musser (1979).

The LIF (4.02 mm) of *Chiropodomys* sp.1 is larger than that of *C. pusillus* (2.70 mm). The TL (91.51 mm) of *Chiropodomys* sp.1 is over 20 mm longer than its HBL (64.70 mm), whereas the TL (81 mm) of *C. pusillus* is only about 5 mm longer than its HBL (76 mm) (Table 1). The dorsal fur of *Chiropodomys* sp.1 is dark brownish‐yellow, similar to that on its sides. In contrast, the dorsal fur of *C. pusillus* is yellowish‐brown and is slightly darker than that of the body sides (Thomas 1893).

Although *C. jingdongensis* is currently listed as a synonym of *C. gliroides* (Musser and Carleton 2005), a morphological comparison with the sympatric *Chiropodomys* sp.1 is still necessary to confirm the taxonomic distinctness of the latter. Distinct morphological differences are evident: in *Chiropodomys* sp.1, the tail hairs are sparse and short, the dorsal fur is dark brownish‐yellow with a narrow black eye‐ring. In contrast, *C. jingdongensis* has dense and long tail hairs, brownish‐yellow fur with a broad black eye‐ring (Wu and Deng 1984).

Compared with all known species of *Chiropoodmys*, the most unique skull feature of *Chiropodomys* sp.1 is that the posterior edge of the incisor foramen reach the anterior edge of the first molar. However, in adults, the posterior edge of the incisor foramen of all known species in the genus *Chiropodomys* do not reach the anterior edge of the first molar.

### 3.2 Phylogentic Relationships

**FIGURE 2:**
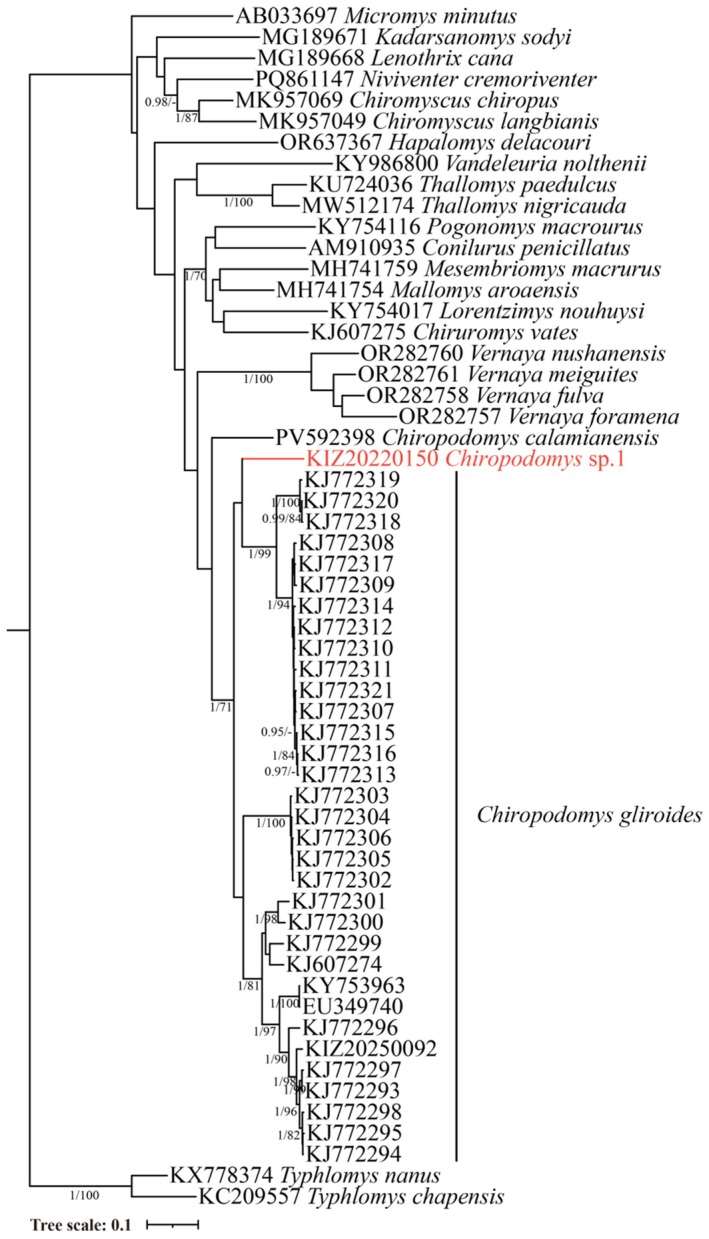
Bayesian phylogenetic tree constructed based on Cyt *b*. The numbers preceding and following the “/” indicate the Bayesian posterior probabilities (values below 0.95 are not shown) and maximum likelihood bootstrap values (values below 70 are not shown), respectively.

In the Bayesian phylogenetic tree constructed based on the Cyt *b* gene, the average standard deviation of split frequencies is 0.005282, the average Potential Scale Reduction Factor (PSRF) is 1.000, and the maximum is 1.005. Regarding the Effective Sample Size (ESS), the minimum ESS occurs for the substitution rate parameter *r*(*C* ↔*T*), with a minimum of 1753.66 and an average of 1819.74. The analysis has reached stationarity and the results are reliable. The phylogenetic tree (Figure 2) reveals that *Chiropodomys* sp.1, *C. calamianensis* , and *C. gliroides* combine together to form a monophyletic group. Within this monophyletic group, *C. gliroides* itself constitutes multiple independent clades, while *Chiropodomys* sp.1 forms a monophyletic group and acts as a sister group relationship with some sequences of *C. gliroides* .

**FIGURE 3:**
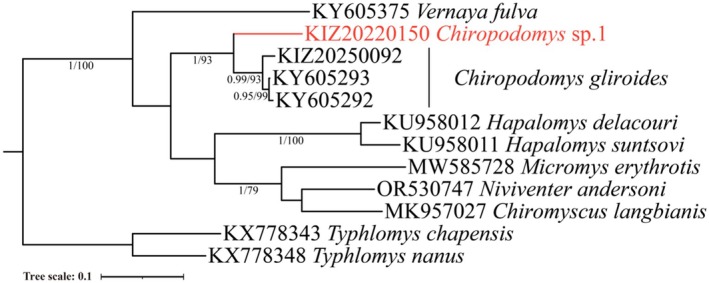
Bayesian phylogenetic tree constructed based on COI. The numbers preceding and following the “/” indicate the Bayesian posterior probabilities (values below 0.95 are not shown) and maximum likelihood bootstrap values (values below 70 are not shown), respectively.

In the Bayesian phylogenetic tree constructed based on the COI gene, the average standard deviation of split frequencies is 0.002657, with a maximum of only 0.008483. The average Potential Scale Reduction Factor (PSRF) is 1.000, and the maximum is 1.001. Regarding the ESS, the minimum ESS occurs for the substitution rate parameter *r*(*C*↔*T*) (minimum ESS = 1383.70, average ESS = 1506.16). The analysis has reached stationarity and the results are reliable. The phylogenetic tree shows that *Chiropodomys* sp.1 forms a monophyletic clade and exhibits a sister group relationship with *C. gliroides* (Figure 3).

### 3.3 Genetic Distance

Genetic distances based on the Cyt *b* sequences revealed that the divergence between *Chiropodomys* sp.1 and *C. gliroides* was 13.06%, and 14.63% between *Chiropodomys* sp.1 and *C. calamianensis* . Distances calculated from the COI sequences showed a divergence of 10.40% between *Chiropodomys* sp.1 and *C. gliroides* (Table 3).

**TABLE 3:** Genetic distances based on Cyt *b* and COI sequences (%).

Cyt *b*				COI		
		1	2			1
1	*Chiropodomys* sp.1			1	*Chiropodomys* sp.1	
2	*C. gliroides*	13.06		2	*C. gliroides*	10.40
3	*C. calamianensis*	14.63	14.61			

## 4 Taxonomic Account

***Chiropodomys wandingensis*** Li & Qian, **sp. nov**. LSID urn: lsid:zoobank.org:act:A4E602A6‐409B‐492E‐9C69‐E8524F373B70

**Holotype**. Adult male; field number KIZ20220150; collection date: 10 July 2019; collected by Shuo Liu.

**Type locality**. Wanding Town, Ruili City, Dehong Dai and Jingpo Autonomous Prefecture, Yunnan Province, China (24.11° N, 98.12° E; elevation: 1174 m).

**Etymology**. The name *wandingensis* refers to the type locality of the species. We propose the English common name “Wanding Pencil‐tailed Tree Mouse” and the Chinese common name “畹町笔尾树鼠” for the newly described species *Chiropodomys wandingensis* sp. nov. This nomenclature reflects both its type locality and the diagnostic pencil‐shaped tail morphology characteristic of the genus *Chiropodomys*.

**Diagnosis**. The back is brownish‐yellow, and the belly is creamy white. The tail is sparsely furnished, with a 2–3 mm tuft of hair at the tip. The incisive foramen is relatively long, and its posterior margin is flush with the anterior edge of the first molar.

**FIGURE 4:**
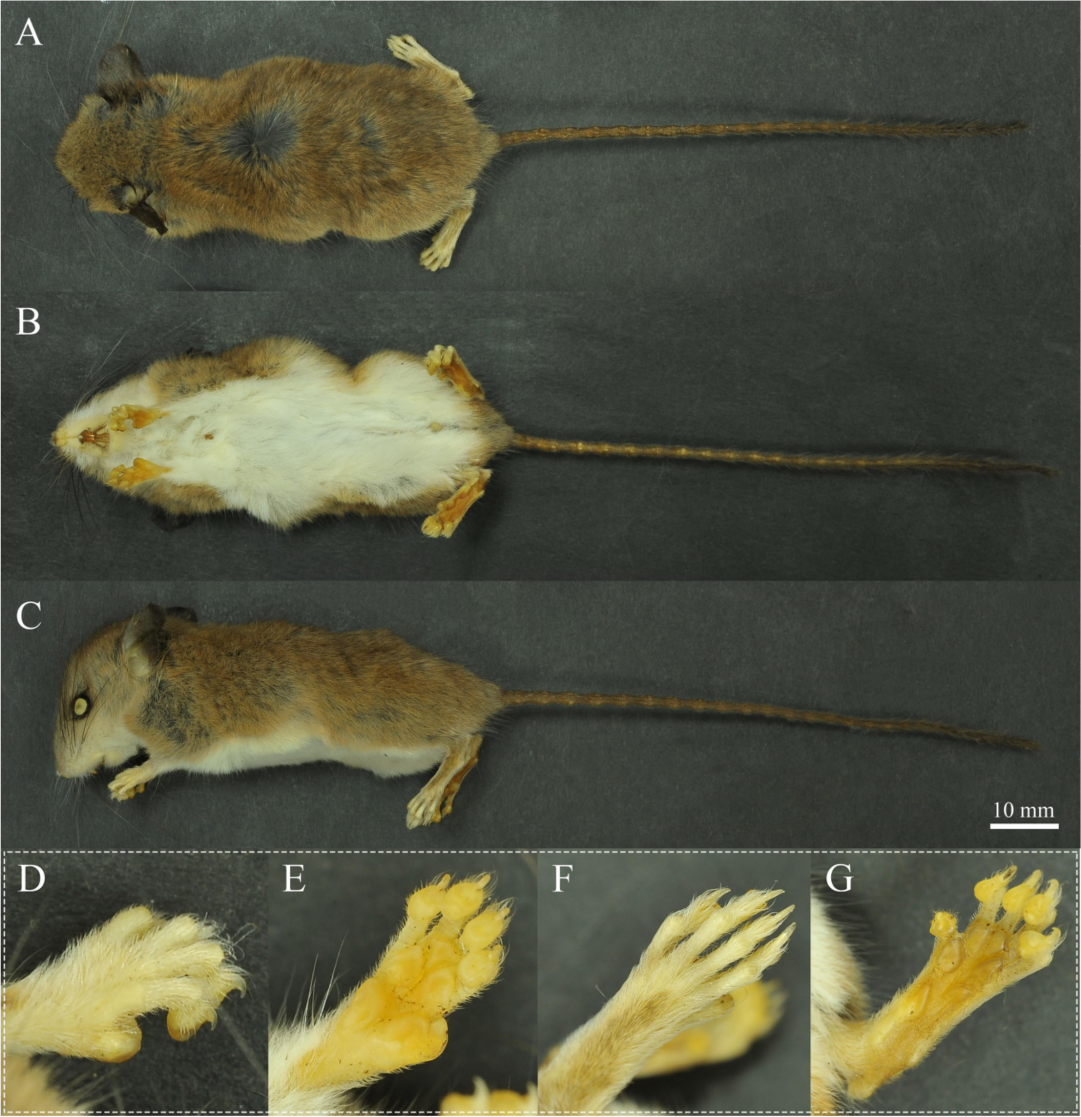
External morphological characteristics of *Chiropodomys wandingensis* sp. nov. (A) Dorsal view of the body; (B) ventral view of the body; (C) lateral view of the body; (D) dorsal view of the forefoot; (E) ventral view of the forefoot; (F) dorsal view of the hindfoot; (G) ventral view of the hindfoot.

**Description**. A small‐sized *Chiropodomys* specimen weighing 9.8 g (recorded after immersion in distilled water with simple dehydration to near dryness). Measurements: HBL 64.70 mm, TL 91.51 mm, HF 15.78 mm, EL 11.36 mm. Dorsal fur shows dark yellowish‐brown coloration, with the upper quarter appearing yellowish‐brown and remaining three‐quarters grayish‐black. Lateral fur appears slightly lighter than ventral fur but maintains overall yellowish‐brown tones. Cheek fur exhibits lighter yellowish‐white coloration. Ventral fur displays creamy white coloration from chin to anus, with sharp demarcation between lateral and ventral areas. The dorsal surfaces of the forefeet, including the digits, display white fur. On the hindlimbs, the dorsal tibial fur matches the lateral body coloration (yellowish‐brown), extending to the middle of the dorsal surface of the hindfoot. Both lateral tibial surfaces and digits exhibit white fur. The white fur on forefeet and hindfeet shows slight yellowish tones, distinct from the pure milky‐white ventral fur (Figure 4).

**FIGURE 5:**
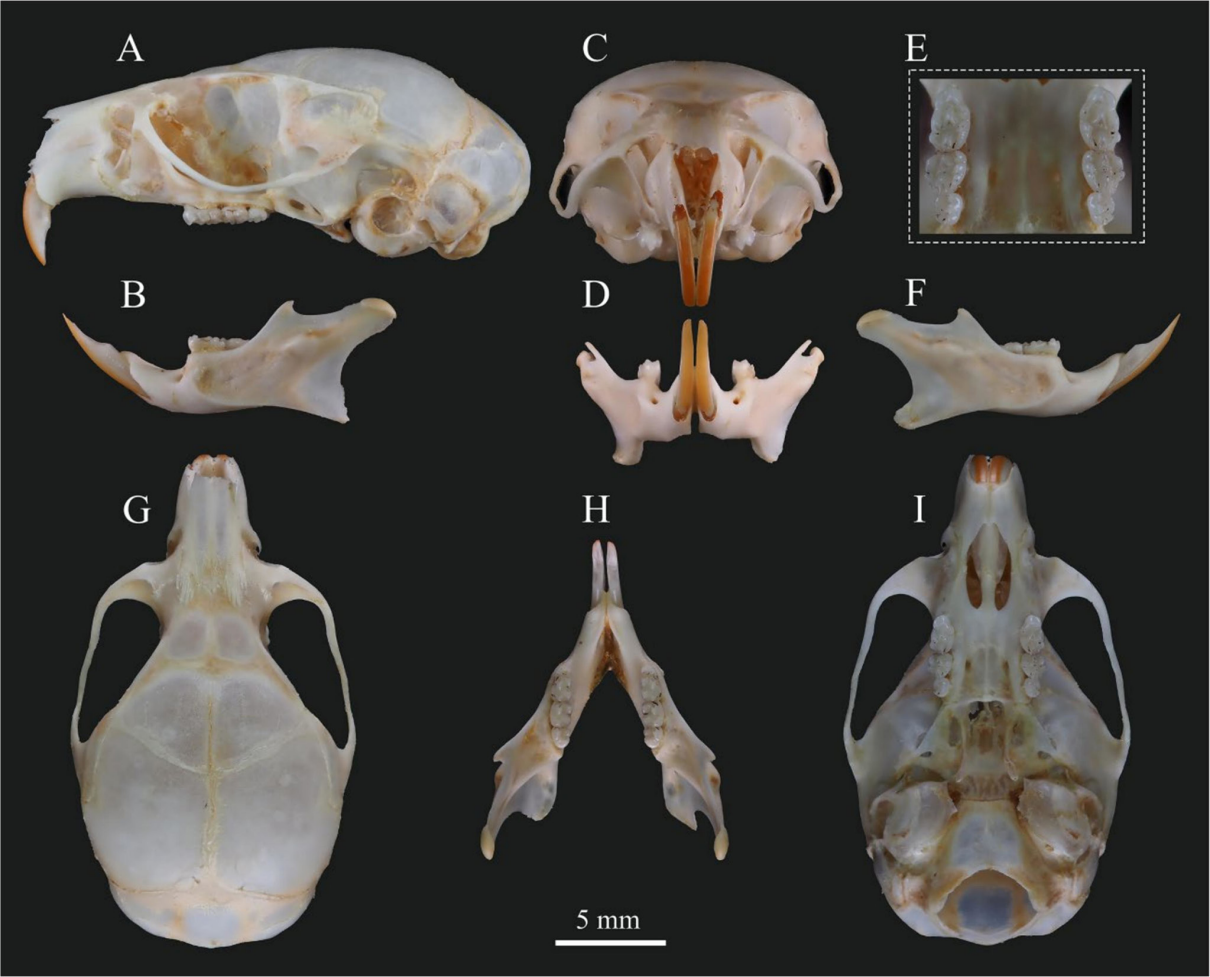
Skull of *Chiropodomys wandingensis* sp. nov. (A) Lateral view of maxilla; (B) lateral view of mandible (right side, broken); (C) anterior view of maxilla; (D) anterior view of mandible; (E) maxillary dentition; (F) lateral view of mandible (left side); (G) ventral view of cranium; (H) occlusal view of mandible; (I) occlusal view of cranium. Mandibular right/left designation follows the cranial anterior view.

The ears are rounded, dark brown on the upper half and pale yellowish‐white on the lower half. A black ring surrounds the eyes. Long black vibrissae originate behind the incisors on each cheek and extend backward beyond the crown and ears. Shorter white vibrissae (about a quarter the length of the black ones) are clustered near the nose. The tail exceeds 40% of the head‐body length, appearing dark brown. The underside of the tail is slightly lighter than the top, but the difference is subtle. Sparse short hairs cover the proximal tail, gradually increasing to 2–3 mm from the middle. These hairs radiate in tufts along the entire tail, with no significant variation in length towards the tip (Figure 5).

The dorsal skull exhibits zygomatic arches arranged in a trapezoidal pattern, narrowing anteriorly and widening posteriorly, while the frontal and parietal bones collectively form a near‐spherical contour. In the maxillary occlusal view, the posterior margin of the incisive foramen aligns horizontally with the anterior margin of the first molar (M^1^), accompanied by a progressive decrease in crown area from M^1^ to M^3^. The pterygoid bone displays near‐parallel anterior borders, diverging laterally in its posterior section (Figure 5).

A lateral perspective reveals anteriorly rounded incisors and a continuous gently ascending slope from the nasal bones to the parietal bones, peaking at the mid‐parietal region. Here, the interparietal and occipital bones create a hemispherical profile, with the zygomatic arch curving downward and the maxillary zygomatic process positioned approximately 1.5 mm superior to the squamosal zygomatic process; the pterygoid bone remains visible in this orientation (Figure 5).

The mandible in lateral view exposes the complete first molar (M^1^) and approximately half of the second molar (M^2^). The coronoid process, triangular in shape, matches the condylar process in height, connected by a smooth transitional junction. The condylar process arcs upward and posteriorly, contrasting with the angular process extending downward and posteriorly. In the mandibular occlusal view, parallel dental rows align with the posteriorly positioned condylar process and laterally flared coronoid process. An anterior skull view distinctly shows orange maxillary incisors and pale yellow mandibular incisors (Figure 5).

**FIGURE 6:**
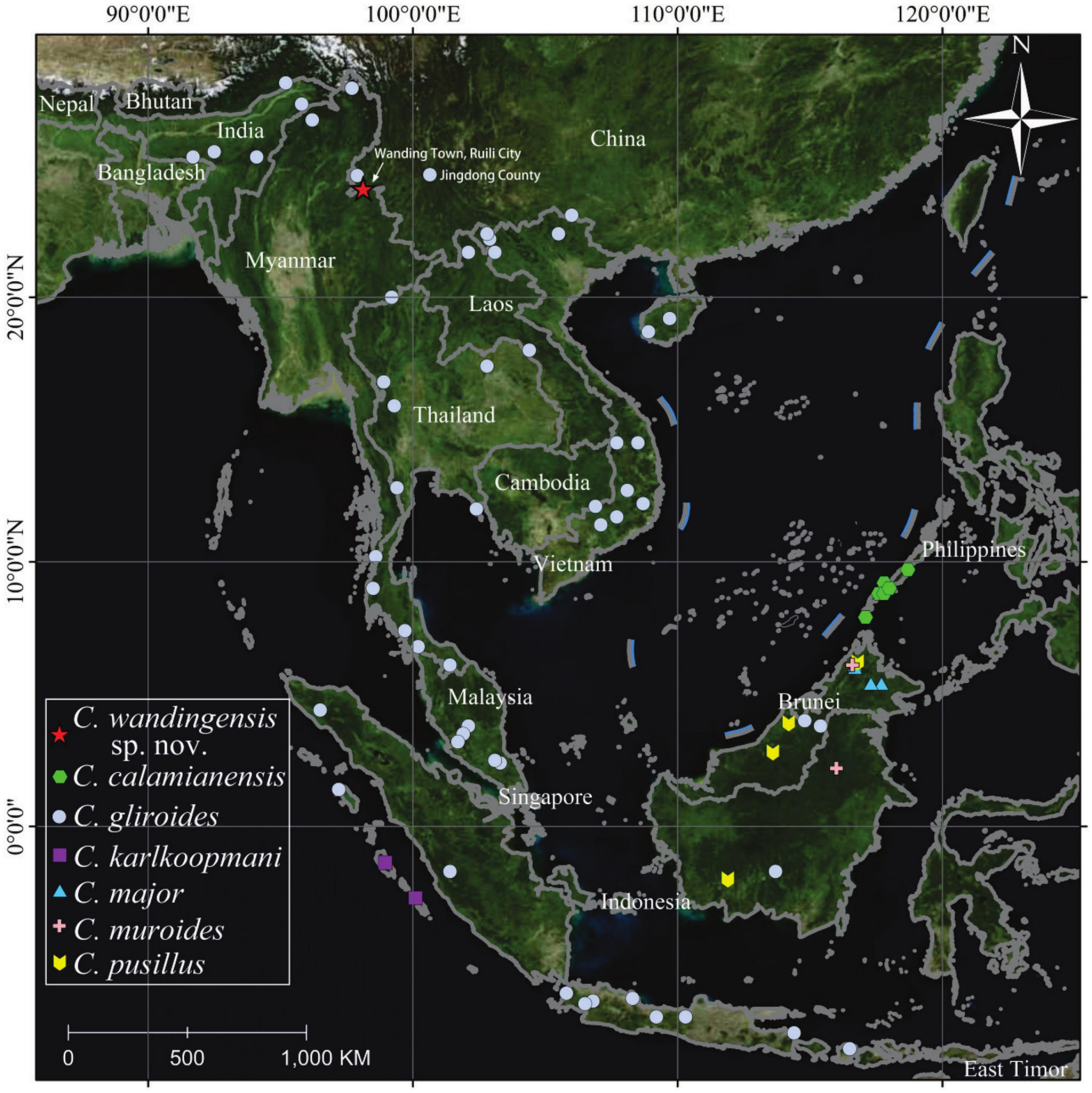
Distribution of Species in the Genus *Chiropodomys* (approval number for base map resource: GS (2025) 1508), distribution range by referring to the distribution data in GBIF (2025) and Musser and Carleton (2005).

**Distribution**. This new species has currently only been discovered in Wanding Town, Ruili City, Yunnan Province, China (Figure 6).

**FIGURE 7:**
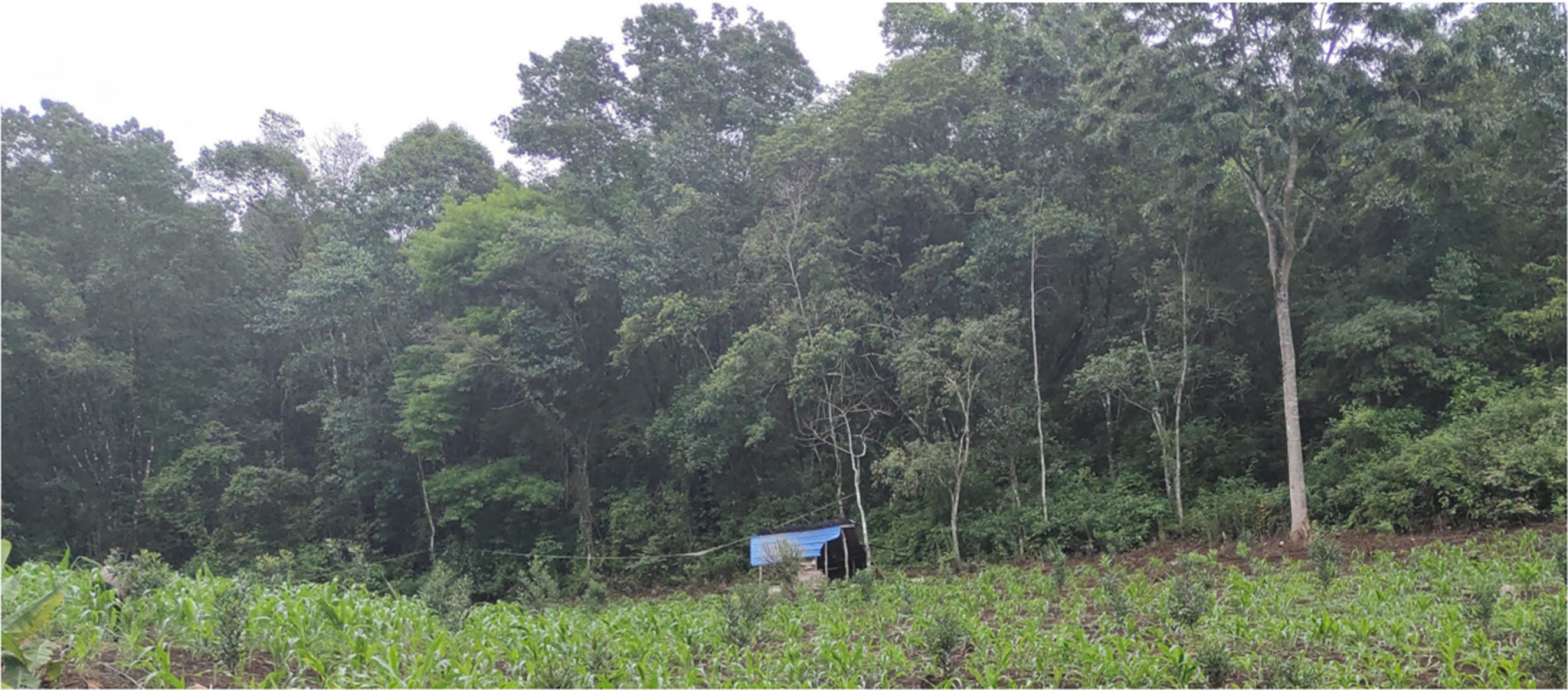
Habitat environment of *Chiropodomys wandingensis* sp. nov. Photographer: Shuo Liu.

**Habitats**. The specimen was collected in a subtropical forest in South Asia, with a monsoon climate; the forest has regrowing trees and thick plants covering the ground. The tree structure is mostly natural, with little damage from human activity (Figure 7). Although the habitat of the newly discovered species *C. wandingensis* sp. nov. is near farmland, the nearby forests are well protected. China has focused on and funded ecological conservation, so these forests show little recent human impact or division. While human actions can threaten small rodents, crops in the fields also offer them food. Also, there are many areas with similar environments across Southeast Asia. This means there could be more undiscovered groups of this species. Although only a single individual has been documented to date, we remain cautiously optimistic about its population viability in the wild.

## 5 Discussion

*Chiropodomys wandingensis* (*Chiropodomys* sp.1, KIZ20220150) can be distinguished from other known species within the genus *Chiropodomys* by the following diagnostic characteristics: the back is brownish‐yellow, the belly is creamy white, the tail is sparsely furnished with a 2–3 mm tuft of hair at the tip, and the incisive foramen is relatively long with its posterior margin flush with the anterior edge of the first molar. Molecular systematics studies reveal that the phylogenetic trees constructed based on mitochondrial Cyt *b* and COI gene fragments both support *Chiropodomys* sp.1 as a monophyletic group. Notably, genetic distance analysis indicates a substantial genetic divergence between *Chiropodomys* sp.1 and its closely related species *C. gliroides* : the genetic distance for Cyt *b* sequences exceeds 13.08%, while that for COI sequences is greater than 10%. Therefore, the available morphological and mitochondrial evidence supports the recognition of this specimen as a distinct species.

Unfortunately, only one specimen represents the new species; it was collected in 2019, and it was accidentally captured on a tree branch in a subtropical forest around 11:00 p.m. In order to add one or two specimens of this new species, although many subsequent efforts have been performed, no additional specimens have been collected so far. This may reflect the species' low population density and highlights the difficulty of its collection. Nevertheless, given the limited number of known species within this genus and the distinct and stable morphological characteristics exhibited by this new species, we consider it crucial to disclose this discovery promptly, as it will not only help to further enrich the species diversity about the genus, but also help to increase public awareness of this extremely rare species.

The diagnostic characters observed in the Wanding specimen include small body size, sparse tail hair, a short terminal tail tuft (2–3 mm), and the posterior margin of the incisive foramen reaching the anterior edge of the first upper molar. These traits could theoretically be affected by individual variation, age, sex, or abnormal cranial development. However, the following facts largely alleviate this concern: (1) The third upper molar is fully developed and shows slight cusp wear. The cranial sutures indicate that the specimen is an adult. This rules out the influence of juvenile or subadult features. (2) The combination of these diagnostic traits clearly exceeds the range of morphological variation observed in available comparative material of known *Chiropodomys* species. Previous studies have not reported such a degree of intraspecific variation (Musser 1979). (3) Mitochondrial genetic distances and phylogenetic analysis also support the morphological differentiation. Therefore, although a single specimen has statistical limitations, the agreement between morphological and molecular evidence is sufficient to distinguish the Wanding specimen from other known species of *Chiropodomys*.

In terms of molecular evidence, this study also has limitations. All genetic data came from mitochondrial genes (COI and Cyt *b*). When specimen numbers are limited, using mitochondrial genes with well‐established reference databases as a preliminary screening tool for species delimitation is a common practice in small mammal taxonomy (Tobe et al. 2010). However, in the genus *Chiropodomys*, publicly available gene sequences are scarce, and most are Cyt *b*. This further restricts the range of comparisons. More importantly, mitochondrial DNA is a single linked locus. It can be affected by factors such as gene introgression and incomplete lineage sorting (Funk and Omland 2003; Galtier et al. 2009; Caraballo et al. 2020), these factors may overestimate or underestimate the true degree of species differentiation. Therefore, the evidence obtained from mitochondrial genetic distances and phylogenetic positions in this paper should be viewed as a supplement and support for the morphological conclusions. It should not be treated as an independent standard for species delimitation.

In the genus *Chiropodomys*, earlier taxonomic changes were based only on morphology. Some originally independent species were merged into *C. gliroides* , and this framework was long followed. One example is *C. pusillus* . Musser (1979) kept it as a subspecies of *C. gliroides* for three reasons. First, the sample size of pusillus was too small for a reliable conclusion. Second, apart from its smaller body size, it showed no unique morphological traits. Third, the body size of *C. pusillus* fits right in with the size gradient seen among rats in this region, from large to small. Therefore, without more morphological and genetic evidence, Musser (1979) adopted a conservative treatment. However, Musser and Carleton (2005) added new morphological evidence. They showed that in most measurements, the few known *C. pusillus* specimens fell outside the range of all other *C. gliroides* samples. Based on this, they recognized *C. pusillus* as a valid species. We agree with this conclusion, but molecular evidence would make it even more reliable.

Another case is *C. jingdongensis* . Musser and Carleton (2005) considered it based on a small sample from western Yunnan, with only a few cranial dimensions averaging slightly larger and no other significant differences. Therefore, they also took a conservative approach and treated *C. jingdongensis* as a synonym of *C. gliroides* . However, we have now found new taxonomic evidence. In this study, specimen KIZ20250092 matches the original description by Wu and Deng (1984) and shows the diagnostic features: similar in size to *C. gliroides* , but with a larger skull and auditory bullae, and the distal half of the tail covered with dense, elongated bristle hairs that fan out laterally like a feather and end in a tufted tip. Its collection site is Jingdong County, Yunnan, the type locality of *C. jingdongensis* . Based on these traits and the location, we confirm that this specimen represents *C. jingdongensis* (currently a synonym of *C. gliroides* ). Wu and Deng (1984) noted that compared to *C. gliroides* , *C. jingdongensis* has especially dense and long bristle hairs on at least the distal half of the tail. Musser (1979) recorded the terminal tail tuft length of *C. gliroides* as 4–5 mm, while that of our specimen KIZ20250092 measures 5–7 mm. At the same time, we have also obtained some interesting molecular findings.

According to Musser (1979), the *C. gliroides* population shows noticeable geographic variation and can be divided into two main types: the Indochinese type and the Malayan type. Musser (1979) believed that although there is some geographical difference between the Indochinese and Malayan types of *C. gliroides* , it is not enough to consider them separate species. Also, within the Indochinese type, most physical features are quite consistent, except for noticeable changes in fur color on the back. However, from a genetic relationship perspective, there appears to be differentiation within the Indochinese type of *C. gliroides* . Meschersky et al. (2016) pointed out that *C. gliroides* in Vietnam, located in the Indochina Peninsula, can be divided into two genetic lineages, north and south, with a genetic distance of more than 10% between them. Unfortunately, Meschersky et al. (2016) did not provide detailed morphological studies. Since there are no overlapping specimens between Musser (1979) and Meschersky et al. (2016), it is difficult to directly link genetic variation with physical features. However, it is worth noting that the specimens of *C. gliroides* in these two studies come from somewhat overlapping geographical areas. This leads to a testable hypothesis: at least in Vietnam, the group identified as *C. gliroides* may not be a single species, but rather a species complex containing hidden species, which earlier classification systems based on stable morphological traits failed to recognize.

Comparing the Cyt *b* sequence of specimen KIZ20250092 with the northern and southern lineages of Vietnamese *C. gliroides* defined by Meschersky et al. (2016), the genetic distance results show: the distance between *C. jingdongensis* and the northern Vietnamese lineage is relatively small (minimum 1.71%), but it differs greatly from the southern Vietnamese lineage (minimum 11.41%) (File S1). This suggests that the species represented by at least part of the northern Vietnamese lineage could be *C. jingdongensis* , while the southern lineage might represent the true *C. gliroides* or a yet undescribed species.

At present, *C. jingdongensis* remains a synonym of *C. gliroides* ; however, our findings suggest that this synonymy may not be fully reliable and that *C. jingdongensis* might represent a distinct species. Given the limited material examined, we can only highlight this noteworthy finding and cannot yet fully resolve its status. We present these observations simply as a record, in the hope that they may prove useful in future taxonomic evaluations.

In summary, this study describes a new species within the genus *Chiropodomys*, *Chiropodomys wandingensis* Li & Qian, sp. nov. Currently, a total of seven species are recognized within the genus globally: *C. wandingensis* sp. nov., *C. gliroides* , *C. major* , *C. pusillus* , *C. karlkoopmani* , *C. muroides* , and *C. calamianensis* . Among these, *C. wandingensis* sp. nov. and *C. gliroides* are distributed in China (Figure 7). The key to the species within the genus is as follows:

**Key to Species of *Chiropodomys***

1. Tail coloration sharply contrasting (proximal one‐third dark brown, distal two‐thirds white).

………………………………………………………………………*C* *. karlkoopmani*

‐ The tail is uniformly colored throughout………………………………2

2. Large body size (head‐body length > 100 mm, tail length > 120 mm)……………………………………………………………………………………3

‐ Small to medium body size (head‐body length < 100 mm, tail length < 120 mm)……………………………………………………………………4

3. Dorsal fur ochraceous‐brown; ventral fur ochraceous‐white to ochraceous‐red, with an ochraceous‐yellow stripe between dorsal and ventral fur…………………………………………… *C. calamianensis*

‐ Dorsal fur grayish‐brown; ventral fur white or creamy, no stripe between dorsal and ventral fur……………………………………*C* *. major*

4. Abdominal hair color buffy dark gray…………………*C* *. muroides*

‐ Abdominal hair color is white………………………………………………5

5. Tail length subequal to head‐body length (tail length/head‐body length < 120%)…………………………………………………*C* *. pusillus*

‐ Tail length markedly exceeding head‐body length (tail length/head‐body length > 120%)………………………………………………………6

6. Tail densely furred………………………………………………*C* *. gliroides*

‐ Tail sparsely furred, terminal hairs 2–3 mm in length……………………………………………………………………………………*C* *. wandingensis*

## Author Contributions

**Yi‐shun Qian:** software (equal), writing – original draft (equal), writing – review and editing (equal). **Xin Mou:** data curation (equal), formal analysis (equal). **Shuo Liu:** investigation (equal). **Song Li:** funding acquisition (equal), methodology (equal), project administration (equal).

## Conflicts of Interest

The authors declare no conflicts of interest.

## Supporting information


**File S1:** The K2P genetic distances calculated based on the Cyt *b* gene.

## Data Availability

The mitochondrial sequence data have been deposited in NCBI; the corresponding accession numbers are listed in Appendix I.

## Appendix I Details of the Sequences Used for Molecular Analysis in This Study

**Table:**

Species name	Cyt *b* accession	Voucher	Locality
*Typhlomys chapensis*	KC209557	Ty‐111	Vietnam
*Typhlomys nanus*	KX778374	KIZ: 0811158 033585	China
*Chiromyscus chiropus*	MK957069	MD10‐15	Vietnam
*Chiromyscus langbianis*	MK957049	16‐010	Vietnam
*Chiruromys vates*	KJ607275	ABTC: 43096	Papua New Guinea
*Conilurus penicillatus*	AM910935	T‐527	Australia
*Hapalomys delacouri*	OR637367	21407	China
*Kadarsanomys sodyi*	MG189671	MZB 33651	Indonesia
*Lenothrix cana*	MG189668	N 92	—
*Lorentzimys nouhuysii*	KY754017	SAMA: 42732	Papua New Guinea
*Mallomys aroaensis*	MH741754	ABTC 45196	Papua New Guinea
*Mesembriomys macrurus*	MH741759	ABTC 07337	Australia
*Micromys minutus*	AB033697	HS1148/KT3068	Japan
*Niviventer cremoriventer*	PQ861147	JAE4777	Indonesia
*Pogonomys macrourus*	KY754116	SAMA: 43144	Papua New Guinea
*Thallomys nigricauda*	MW512174	ANG0157	Angola
*Thallomys paedulcus*	KU724036	TM41047	RSA
*Vandeleuria nolthenii*	KY986800	WHT: 6940	Sri Lanka
*Vernaya foramena*	OR282757	SAF20233	China
*Vernaya fulva*	OR282758	SCNU02747	China
*Vernaya meiguites*	OR282761	SAF220266	China
*Vernaya nushanensis*	OR282760	XMS170115	China
*Chiropodomys calamianensis*	PV592398	FMNH:195289	—
*Chiropodomys gliroides*	KJ772293	ZIN99863	Vietnam: Lao Cai Province
*Chiropodomys gliroides*	KJ772300	ZIN91159	Vietnam: Kon Tum Province
*Chiropodomys gliroides*	KJ772303	ZIN97722	Vietnam: Lam Dong Province
*Chiropodomys gliroides*	KJ772305	ZIN97728	Vietnam: Lam Dong Province
*Chiropodomys gliroides*	KJ772309	ZIN99467	Vietnam: Binh Phuoc Province
*Chiropodomys gliroides*	KJ772316	ZIN99473	Vietnam: Dong Nai Province
*Chiropodomys gliroides*	KY753963	AMCC: 101511	Vietnam: Ha Giang Province
*Chiropodomys gliroides*	KJ772294	ZIN99864	Vietnam: Lao Cai Province
*Chiropodomys gliroides*	KJ772298	ZIN101547	Vietnam: Lao Cai Province
*Chiropodomys gliroides*	KJ772297	ZIN101546	Vietnam: Lao Cai Province
*Chiropodomys gliroides*	KJ772295	ZIN101542	Vietnam: Lao Cai Province
*Chiropodomys gliroides*	KJ772296	ZIN101543	Vietnam: Lao Cai Province
*Chiropodomys gliroides*	KJ607274	FMNH: 212935	—
*Chiropodomys gliroides*	KJ772299	ZIN98662	Laos: Khammouane Province
*Chiropodomys gliroides*	KJ772301	ZIN91160	Vietnam: Kon Tum Province
*Chiropodomys gliroides*	KJ772304	ZIN97717	Vietnam: Lam Dong Province
*Chiropodomys gliroides*	KJ772302	ZIN97725	Vietnam: Lam Dong Province
*Chiropodomys gliroides*	KJ772306	ZIN97721	Vietnam: Lam Dong Province
*Chiropodomys gliroides*	KJ772319	ZIN101292	Vietnam: Lam Dong Province
*Chiropodomys gliroides*	KJ772318	ZIN101291	Vietnam: Lam Dong Province
*Chiropodomys gliroides*	KJ772320	ZIN101293	Vietnam: Lam Dong Province
*Chiropodomys gliroides*	KJ772317	S186543	Vietnam: Dong Nai Province
*Chiropodomys gliroides*	KJ772310	ZIN99470	Vietnam: Binh Phuoc Province
*Chiropodomys gliroides*	KJ772312	ZIN99472	Vietnam: Dong Nai Province
*Chiropodomys gliroides*	KJ772308	ZIN98921	Vietnam: Binh Phuoc Province
*Chiropodomys gliroides*	KJ772311	ZIN99931	Vietnam: Dong Nai Province
*Chiropodomys gliroides*	KJ772307	ZIN98920	Vietnam: Binh Phuoc Province
*Chiropodomys gliroides*	KJ772313	ZIN99930	Vietnam: Dong Nai Province
*Chiropodomys gliroides*	KJ772321	ZIN99039	Vietnam: Dong Nai Province
*Chiropodomys gliroides*	KJ772314	ZIN99474	Vietnam: Dong Nai Province
*Chiropodomys gliroides*	KJ772315	S186541	Vietnam: Dong Nai Province
*Chiropodomys gliroides*	EU349740	AMCC 101511	—
*Chiropodomys* sp.1	PZ622142	KIZ20220150	China: Yunnan
*Chiropodomys gliroides*	PZ622143	KIZ20250092	China: Yunnan

Species name	COI accession	Voucher	Locality
*Chiromyscus langbianis*	MK957027	16‐036	Vietnam
*Hapalomys suntsovi*	KU958011	ZMMU S‐186111	Vietnam
*Hapalomys delacouri*	KU958012	ZIN 103040	Vietnam
*Micromys erythrotis*	MW585728	QLF1911340	China
*Niviventer andersoni*	OR530747	USNM: MAMM: 574355	China
*Vernaya fulva*	KY605375	LC027	—
*Typhlomys chapensis*	KX778343	KIZ:AL1311475 029295	China
*Typhlomys nanus*	KX778348	KIZ:1112274 028336	China
*Chiropodomys gliroides*	KY605293	LC113	—
*Chiropodomys gliroides*	KY605292	LC033	—
*Chiropodomys* sp.1	PZ629148	KIZ20220150	China: Yunnan
*Chiropodomys gliroides*	PZ629149	KIZ20250092	China: Yunnan
